# Seroprevalence of *Toxoplasma gondii* infection among pregnant women in Windhoek, Namibia, in 2016

**DOI:** 10.4102/sajid.v35i1.25

**Published:** 2020-05-13

**Authors:** Berta E. van der Colf, Gert U. van Zyl, Bruce H. Noden, Dismas Ntirampeba

**Affiliations:** 1 Department of Health Sciences, Faculty of Health and Applied Sciences, Namibia University of Science and Technology, Windhoek, Namibia; 2 Division of Medical Virology, Faculty of Medicine and Health Sciences, Stellenbosch University, Cape Town, South Africa; 3 Department of Entomology and Plant Pathology, Division of Agricultural Sciences and Natural Resources in the College of Agricultural Sciences and Natural Resources, Oklahoma State University, Stillwater, United States; 4 Department of Mathematics and Statistics, Faculty of Health and Applied Sciences, Namibia University of Science and Technology, Windhoek, Namibia

**Keywords:** *Toxoplasma gondii*, toxoplasmosis, seroprevalence, IgG avidity, pregnant women, Namibia

## Abstract

**Background:**

When a pregnant woman contracts *Toxoplasma gondii* (*T. gondii*) infection during pregnancy, it may be vertically transmitted to the foetus. Information on the incidence of congenital toxoplasmosis (CT) in developing countries is scarce. Most studies focus on the seroprevalence of *T. gondii* infection among pregnant women. This study aimed to determine the seroprevalence of *T. gondii* infection among pregnant women attending public antenatal care in Windhoek, Namibia, in 2016.

**Methods:**

In this descriptive study, 344 urban pregnant women attending public antenatal care were voluntarily enrolled in the study. Seroprevalence of anti-*T. gondii* Immunoglobulin G (IgG) was determined by automated immunoassay. Samples with a positive *T. gondii* IgG result were tested for *T. gondii* Immunoglobulin M (IgM) and specific IgG avidity by using an enzyme-linked immunosorbent assay (ELISA) test. A questionnaire captured demographic data and exposure to risk factors. Data were analysed using Statistical Package for the Social Sciences (SPSS) and R.

**Results:**

Anti-*T. gondii* IgG was found in nine (2.61%) pregnant women. There was no association of anti-*T. gondii* IgG with demographic characteristics or exposure to risk factors.Anti-*T. gondii* IgM was positive in one (0.3%) woman, while three (0.9%) women had borderline anti-*T. gondii* IgM results. Specific IgG avidity was low, equivocal and high in 0%, 33% and 67% of seropositive pregnant women, respectively.

**Conclusion:**

Seroprevalence of anti-*T. gondii* IgG is much lower in Namibia than is reported in other developing countries. Investigation into specific IgM seropositivity and IgG avidity showed that pregnant women in the central region of Namibia are at low risk of vertical transmission and development of CT.

## Introduction

*Toxoplasma gondii* (*T. gondii*) is a protozoan parasite with the only definitive hosts being the cat species. Infection occurs via contamination of soil and water, and the parasite can be found in all warm-blooded vertebrates and some reptiles. Humans are intermediate hosts. Infections in humans occur through the ingestion of raw or undercooked meat, unpasteurised milk or contaminated water or by sharing the environment with cats and inadvertently ingesting oocytes from cat faeces. Toxoplasmosis can be transmitted from a mother to a foetus and can cause deafness, blindness, mental retardation, physical impairment or even stillbirth. If a pregnant woman is diagnosed with *T. gondii* infection, antimicrobial treatment is possible, although the success of the treatment will depend on a number of variables like the dose and the route of administration.^1^ Congenital toxoplasmosis (CT) may occur in spite of treatment with spiramycin of pregnant women with primary *T. gondii* infection.^2^ Furthermore, spiramycin is not available in the public or the private sector in Namibia.

### Congenital toxoplasmosis

Immunocompetent and immunocompromised women contracting *T. gondii* infection for the first time during pregnancy run a risk of transmitting the infection to the foetus because of the parasite crossing the placenta from the maternal blood. A host that is immunocompetent acquires life-long immunity against toxoplasmosis when infected with *T. gondii*. A new *T. gondii* infection contracted 4–6 months before conception will usually not result in mother-to-child transmission of toxoplasmosis on subsequent exposures. In immunocompromised women who are positive for human immunodeficiency virus (HIV), *T. gondii* infection may reactivate with resulting vertical transmission.^3,4^ In a study in Brazil, 28% of *T. gondii* Immunoglobulin M (IgM)-positive pregnant women with unknown HIV status transmitted the infection vertically.^2^

Only about 30% of infants born to mothers who had seroconversion in pregnancy acquire prenatal infection. If the infection in a pregnant woman is not treated, the risk of intrauterine infection increases with gestational age, that is, from 14% for primary maternal infection in the first trimester to 59% for primary maternal infection in the last trimester.^3^ When the primary infection occurs during advanced gestation, the risk of transmission to the foetus is higher. However, when transmission occurs early in gestation, the risk of symptomatic infection and severe disease is high.^5^ Clinical signs of toxoplasmosis in neonates include the classical triad, namely, intracranial calcification, hydrocephalus and retinochoroiditis. About 10% of prenatal infections result in abortion or neonatal death. The surviving infants suffer from progressive neurologic complications like mental retardation, learning problems, hearing and visual impairment or seizures and have special care and education needs. Children who have been infected before birth might be asymptomatic at birth but may develop complications associated with toxoplasmosis later in life.^3,6,7^ Clinical trials indicate that early treatment of these children can decrease manifestations of disease and improve the quality of life.^3^

Congenital toxoplasmosis can best be prevented by ensuring that pregnant women who are seronegative do not get infected, or by treatment of pregnant women who are diagnosed with a primary infection, as evident from seroconversion, T. *gondii* Immunoglobulin M (IgM) positivity and low Immunoglobulin G (IgG) avidity. Screening programmes for toxoplasmosis have been introduced in many European countries and in the United States. These include either diagnosis of primary maternal infection with *T. gondii* during pregnancy or detection of congenital infections in neonates at birth.^3^ No routine screening for *T. gondii* is done in Namibia, and only two previous studies were conducted to determine the prevalence of toxoplasmosis among blood donors.^8,9^ The aim of this study was to determine the prevalence of antibodies to *T. gondii* among urban pregnant women attending public antenatal care in Windhoek, Namibia, from September to October 2016.

## Materials and methods

### Study design

This was a descriptive study aiming to describe the seroprevalence of *T. gondii* among pregnant women in Windhoek.

### Sample size

At an expected prevalence of 6% of *T. gondii* infection, using Cochran’s formula, a sample size of 542 would provide a precision (error margin, above and below the prevalence estimate) of 2%. Estimated prevalence was based on two previous publications in Namibia and the seroprevalence in neighbouring South Africa. Funding was available to enrol 344 participants in the study.

### Exclusion and inclusion criteria

All pregnant women who attended public antenatal care in Windhoek, from 12 September to 11 October 2016, were informed about the study, and all women who were identified by the clinic to have their blood drawn for routine antenatal testing were eligible to participate. Five participants were excluded because of not signing the consent form by omission and one Ovahimba woman was excluded because of not understanding English or Afrikaans.

### Sample collection

Participation in the study was voluntary, and written consent was obtained from each participant and guardian, where applicable. A venous blood sample was collected from each consenting participant and transported immediately to the testing laboratory. A questionnaire was administered to collect information on demographic characteristics and exposure to risk factors, and was filled by consenting participants. Questions were derived from the literature.

### Sample storage

Blood samples were transported to the laboratory within an hour. Serum was stored for not more than a week at 2ºC – 8ºC and then at -80ºC until testing.

### Laboratory methods

An automated chemiluminescent microparticle immunoassay was performed in an ISO 15189 accredited laboratory to determine seroprevalence of IgG antibodies to *T. gondii* (access immunoassay systems, Beckman Coulter Eurocenter SA, 22 rue Juste-Olivier, Case Postale 1044, CH – 1260 Nyon 1, Switzerland). The presence of anti-*T. gondii* IgM antibodies was determined in samples testing positive for specific IgG using the ELISA method (Euroimmun Medizinische Labordiagnostika, D-23560 Lubeck, Seekamp 31, Germany). Cut-off points were calculated by using the ratio of the extinction of the control or patient sample to the extinction of the calibrator. The extinction value of the calibrator defined the cut-off value as recommended by the manufacturer. IgG avidity was determined in samples testing positive for specific IgG by performing a manual ELISA test with urea neutralisation (Euroimmun).

For interpretation of IgG results, recommendations from the manufacturer were followed. Specimens with specific IgG concentration of > 10.5 IU/mL were considered positive for *T. gondii* past or current infection. Acute infection with *T. gondii* was determined by the presence of anti-*T. gondii* IgM antibodies, and specific IgG antibodies with low avidity. Recommendations from the manufacturer were followed for interpretation of results. For interpretation of IgM results, an extinction ratio (sample relative to calibrator) of <0.8 was considered negative, ≥0.8 to <1.1 was borderline and ≥1.1 was considered positive. For interpretation of avidity results, the ratio of the enzyme immunoassay optical density when urea-treated relative to untreated was used. Low avidity was defined as a ratio of <40%, which was indicative of acute *T. gondii* infection. A ratio of 40% – 60% was considered equivocal, while a ratio above 60% was an indication of high avidity antibodies.

### Data analysis

Association of demographic characteristics (maternal age, gestational age and parity) with seroprevalence of *T. gondii* infection among pregnant women was investigated. Association of risk factors (water source, living with cats or dogs, blood transfusion, as well as consumption of untreated water, unpasteurised cow’s milk, goat’s milk, raw or undercooked meat or unwashed fruit/vegetables) with seroprevalence of *T. gondii* infection was investigated. A case-based description of anti-*T. gondii* IgG and IgM seropositivity and IgG avidity was provided for nine participants who tested IgG positive. Seropositivity among five age groups was evaluated with the assumption that all age groups had had similar exposure to *T. gondii*. All data were entered into an Excel spreadsheet (Microsoft Corporation, One Microsoft Way, Redmond, WA 98052-7329) and analysed using Statistical Package for the Social Sciences statistical software (version 24, IBM North America, 590 Madison Avenue, New York, NY 10022) and R version 3.2.2.^10^ Chi-square test and Fisher’s exact test (when the expected frequency in any cell of the two-by-two table was less than 5) were used to assess associations between population characteristics provided in the surveys and seropositivity results. Multivariate analysis was performed with generalised linear models using binary logistic regression. Beta values were used to calculate odds ratios of variables, while the Wald 95% confidence intervals gave an indication of the width of the odds ratios; *p-*values less than 0.05 were considered statistically significant.

### Ethical consideration

Ethical approval to conduct the study was obtained from the School of Health and Applied Sciences, Polytechnic of Namibia; the Ministry of Health and Social Services in Namibia; and the Health Research Ethics Committee at Stellenbosch University, Cape Town, South Africa.

## Results

A total of 344 pregnant women donated a blood sample and filled the questionnaire. Maternal age at enrolment ranged between 17 and 47 years with the median age of 27 years. All but two women were residing in an urban area. Forty-one women (11.9%) were in the first trimester, 172 (50%) in the second trimester and 130 (37.8%) in the third trimester of pregnancy during enrolment in the study. A total of 129 women (37.5%) were primigravidae, 100 (29.1%) had one previous pregnancy, while 115 (33.4%) had had two or more previous pregnancies.

The overall seroprevalence of anti-*T. gondii* IgG among pregnant women attending public antenatal care in Windhoek was 2.61% (Table 1). The overall susceptibility to *T. gondii* infection was high (*n* = 335; 97.4%). There was no statistically significant difference between seroprevalence of anti-*T. gondii* IgG in different age groups. There was no statistically significant difference between the occurrences of anti-*T. gondii* IgG in different trimesters of pregnancy. There was no statistically significant difference between the occurrences of anti-*T. gondii* IgG in different parities although the highest observed seroprevalence was in primigravida cases.

**TABLE 1:** Association of demographic characteristics with seroprevalence of *Toxoplasma gondii* among urban pregnant women (***N*** = 344) attending public antenatal care in Windhoek, Namibia, 2016.

Demographic characteristic	*N*	IgG +†		Bivariate *p*-value	Multivariate *p*-value	Odds ratio‡	95% CI§
		*n*	%				
**Overall**	344	9	2.61	-	-	-	-
**Maternal age**							
15‒20	38	0	0.00	0.383	0.211	1.08	−0.046–0.209
21‒25	108	3	2.78	-	-	-	-
26‒30	96	5	5.21	-	-	-	-
31‒35	59	0	0.00	-	-	-	-
36‒47	43	1	2.33	-	-	-	-
**Gestational age**							
1st trimester	41	0	0.00	0.793	0.999	N/A¶	-
2nd trimester	172	5	2.91	-	0.796	0.84	−1.533‒1.176
3rd trimester	130	4	3.08	-	-	-	-
**Primigravida**	129	6	4.65	0.220	0.048	12.16	0.022‒4.973
1 previous pregnancy	100	2	2.00	-	0.334	3.41	−1.262‒3.713
≥ 2 previous pregnancies	115	1	0.87	-	-	-	-

† , Including borderline positive results;

‡ , Adjusted odds ratio was not calculated since no predictor was statistically significant;

§ , Ninety-five percent Wald confidence interval of the odds ratio;

¶ , Not available since one predictor equals zero.

N/A, not applicable.

This study found no association between risk factors and seropositivity of anti-*T. gondii* IgG in pregnant women (Table 2). Results showed that none of own tap versus communal tap as water source, living with cats or dogs, or blood transfusion was associated with seroprevalence of *T. gondii* infection. Consumption of water from a dam or river; unpasteurised cow’s milk and goat’s milk; raw/undercooked meat or unwashed fruit/vegetables were not identified as risk factors for *T. gondii* exposure.

**TABLE 2:** Association of risk factors with seroprevalence of *Toxoplasma gondii* among urban pregnant women (*N* = 344) attending public antenatal care in Windhoek, Namibia, 2016.

Risk factor	*N*	IgG +†		Bivariate *p*-value	Multivariate *p*-value	Odds ratio‡	95% CI§
		*n*	*%*				
**Water source**							
Own tap	226	9	3.98	0.185	0.998	N/A¶	-
Communal tap	114	0	0.00	-	-	-	-
**Live with cats**							
Yes	45	0	0.00	0.612	0.999	N/A	-
No	299	9	3.01	-	-	-	-
**Live with dogs**							
Yes	122	4	3.28	0.726	0.787	1.24	−1.349−1.779
No	222	5	2.25	-	-	-	-
**Received a blood transfusion**							
Yes	6	1	16.67	0.172	0.417	3.55	−1.794−4.331
No	327	8	2.45	-	-	-	-
**Consumption**							
**Water from dam or river**							
Yes	55	3	5.45	0.160	0.088	4.75	−0.235−3.352
No	289	6	2.08	-	-	-	-
**Unpasteurized cow’s milk**							
Yes	39	3	7.69	0.070	0.052	7.30	−0.017−3.992
No	305	6	1.97	-	-	-	-
**Goat’s milk**							
Yes	7	1	14.29	0.171	0.703	1.87	−2.590−3.838
No	337	8	2.37	-	-	-	-
**Raw or undercooked meat**							
Yes	97	2	2.06	1.000	0.323	2.71	−0.981−2.972
No	247	7	2.83	-	-	-	-
**Unwashed fruit and/or vegetables**							
Yes	83	0	0	0.121	0.998	N/A	-
No	261	9	3.45	-	-	-	-

† , Including borderline positive results;

‡ , Adjusted odds ratio was not calculated since no predictor was statistically significant;

§ , Ninety-five percent Wald confidence interval of the odds ratio;

¶ , Not available since one predictor equals zero.

N/A, not applicable.

Of the 344 participants, nine (2.61%) had a positive anti-*T. gondii* IgG result, while one (0.3%) had a positive IgM result and three (0.9%) had a borderline-positive IgM result (Table 3). The mean anti-*T. gondii* IgG titre among *T. gondii*-positive pregnant women was 132.1 IU/mL, ranging between 25.9 IU/mL and 298.2 IU/mL. Specific IgG avidity among infected women was low, equivocal and high in 0%, 33% and 67% of positive IgG cases, respectively.

**TABLE 3:** Anti-*Toxoplasma gondii* IgG and IgM seropositivity and IgG avidity among pregnant women.

Case	IgG IU/mL	IgM	IgG avidity
5015	38.8	Neg	High
5022	298.2	Neg	High
5040	168.2	Pos	Equivocal
5050	144.2	Borderline	Equivocal
5116	25.9	Borderline	Equivocal
5145	210.5	Borderline	High
5164	83.3	Neg	High
5320	113.6	Neg	High
5328	106.4	Neg	High

Neg, negative; Pos, positive; IgG, Immunoglobulin G; IgM, Immunoglobulin M.

## Discussion

### Seroprevalence of anti-Toxoplasma gondii IgG

Very low seroprevalence of anti-*T. gondii* IgG (2.61%) was found among urban pregnant women attending public antenatal care in Windhoek, in 2016. This is comparable to a previous study in Namibia which found 1% seroprevalence of anti-*T. gondii* among blood donors.^9^ Possible reasons for this low seroprevalence could be the arid climate and the high altitude of the capital city, Windhoek.^11^ Oocysts cannot withstand dry conditions that prevail during extended periods of drought in central Namibia. The worst drought, since recording of rainfall had started, occurred in Namibia in 2016. The seroprevalence of anti-*T. gondii* found in 2016 was lower than the 12.1% recorded among blood donors in Windhoek in 1978.^8^ This finding is in line with the worldwide trend of decreasing seroprevalence of anti-*T. gondii* over the previous decades.^12^ Factors that could contribute to the decreasing trend are urbanisation, which is accompanied by better access to safe drinking water and abattoirs, as well as increased use of pasteurised cow’s milk.

Being the driest African country south of the Sahara, Namibia shows much lower seroprevalence of *T. gondii* infection than in other developing countries (Table 4).^8,13,14,15,16,17,18,19,20,21,22,23,24,25,26,27,28^ It is however comparable to seroprevalence in neighbouring South Africa, with which Namibia shares weather patterns.^23^ The seroprevalence is the same as in Mexico, which has a similar arid climate.^29^ A review of studies conducted in Africa found a median seroprevalence of *T. gondii* of 37%, with rates ranging from 6.4% to 74.5%.^12^ Very little is known about the burden of CT in developing countries. In Brazil, Neto et al. found 0.06% of neonates being anti-*T. gondii* IgG seropositive.^30^ It is postulated that the high burden of CT in South America can be attributed to more pathogenic genotypes that circulate in that part of the world.^31^

**TABLE 4:** Seroprevalence of *Toxoplasma gondii* in developing countries.

Country	Year published	IgG seroprevalence (%)	Population	*N*	Method	Reference
Namibia	1978	12	Blood donors	261	IFA	Jacobs^8^
Sudan	1991	41.7	General population	386	LAT	Abdel-Hameed^13^
Benin	1995	53.6	Pregnant women	211	ELISA	Rodier^14^
Tanzania	1995	35	Pregnant women	849	Sabin-Feldman dye test	Doehring^15^
India	2004	IgG 45 IgM 3.3 1.1 low avidity	Pregnant women	180	ELISA	Singh^16^
Ethiopia	2007	60	Urban population	65	MDAT	Negash^17^
Nigeria	2007	20.8	Healthy individuals	144	-	Uneke^18^
Tanzania	2009	46	Occupationally exposed	199	LAT	Swai^19^
Brazil	2010	IgG 62 IgM 3.4	Pregnant women	574	Patient records	Dos Santos Goncalves^20^
Gabon	2010	56	Pregnant women	839	ELFA	Mickoto^21^
Mozambique	2010	18.7	Pregnant women	150	ELISA	Sitoe^22^
South Africa	2011	6.4	General population	376	LAT	Kistiah^23^
Nigeria	2011	IgG 32.6 IgM 7.6	Pregnant women	276	ELISA	Deji-Agboola^24^
Egypt	2012	IgG 67.5 IgM 2.8 0.3 low avidity	Pregnant women	323	ELFA	El Deeb^25^
Benin	2014	IgG 30 IgM 0.4	Pregnant women	283	ELISA	De Paschale^26^
Congo	2014	IgG 80.3 IgM 4.4 11.8 low avidity	Pregnant women	781	ELFA	Doudou^27^
Namibia	2014	1.0	Blood donors	320	ELISA	Van der Colf^9^
Ethiopia	2015	18.5	Pregnant women	384	LAT	Awoke^28^
Mexico	2018	3.6	Women of reproductive age	445	ELISA	Alvarado-Esquivel^29^

Note: Please see the full reference list of the article, Van der Colf BE, Van Zyl GU, Noden BH, Ntirampeba D. Seroprevalence of Toxoplasma gondii infection among pregnant women in Windhoek, Namibia, in 2016. S Afr J Infect Dis. 2020;35(1), a25. https://doi.org/10.4102/sajid.v35i1.25, for more information.

IgG, Immunoglobulin G; IgM, Immunoglobulin M, IFA, Immunofluorescence Assay; LAT, Latex agglutination test; ELISA, Enzyme-linked immunosorbent assay; MDAT, Agglutination test; ELFA, Enzyme-linked fluorescence assay.

In Windhoek, 97.4% of pregnant women attending public antenatal care remain susceptible to *T. gondii*, posing a risk of vertical transmission and CT if they get infected while pregnant. Infection pressure is, however, low – a large population is at risk, but the probability of exposure is low. In hyperendemic settings, acquisition and immunity are likely before women get pregnant, whereas models suggest that when the incidence of *T. gondii* infection among pregnant women is 4% per annum, the risk of seroconversion during pregnancy and occurrence of CT would be the highest. This correlates with a susceptibility of 40% among pregnant women, which is much lower than the susceptibility found in this study.^31^

### Factors associated with seropositivity

There was no significant difference in prevalence of anti-*T. gondii* IgG among the five different age groups of pregnant women. Seroprevalence linearly increased in the lowest three age groups (15–20 years, 21–25 years and 26–30 years), only to decrease in older women (31–47 years) (Table 1). Seroprevalence of anti-*T. gondii* IgG increased with gestational age although the difference was not statistically significant. Primigravidae showed the highest seroprevalence of anti-*T. gondii* IgG, which is in line with the finding of higher seroprevalence among younger women.

There was no significant association between risk factors and seroprevalence of *T. gondii* (Table 2). Surprisingly, participants with access to own tap showed the highest seroprevalence of *T. gondii*. However, this association was not significant. Another interesting finding was the lack of association between consumption of unwashed fruit or vegetables and seropositivity, while 3.45% of participants denied that this consumption showed seropositivity of *T. gondii* infection. Alvarado-Esquivel et al. reported a similar finding.^29^ These exposures to *T. gondii* could possibly be because of other risk factors like working with raw meat, which were not assessed in our study.

Magwedere et al. included a discussion on *T. gondii* in their report on zoonoses posing a possible threat to the wildlife industry in Namibia.^32^ Working with raw game meat or consumption of undercooked game meat could possibly pose a risk of exposure to *T. gondii*. A previous study found presence of *T. gondii* in lions in Namibia. Among a pride of five lions in a private reserve, four showed evidence of *T. gondii* infection.^33^ A high prevalence of *T. gondii* was also found in feral cats in the Western Cape Province in South Africa, providing a reservoir for the parasite and a potential source of contamination of the environment.^34^ In the 1990s, a municipality in the Province of British Columbia in Western Canada experienced an outbreak of toxoplasmosis, with the municipal water supply implicated as the source of infection.^35,36^

### Specific IgM and IgG avidity

Very few pregnant women had possibly been infected during pregnancy; therefore, the risk of giving birth to an infant with CT was minimal. Anti-*T. gondii* IgM antibodies and specific IgG avidity were tested only in samples positive for anti-*T. gondii* IgG. Other studies found very low prevalence of samples positive for anti-*T. gondii* IgM only (0.3%,^37^ 0.9%^38^), so the possibility of finding IgM positive while IgG is negative could be negated.

It can thus be concluded that the pregnant women who presented with anti-*T. gondii* IgG antibodies were infected before they became pregnant, with low risk of vertical transmission. Avidity testing is widely used in Europe and increasingly in southern Africa. The French National Reference Center for Toxoplasmosis has proposed algorithms for interpretation of serologic testing (IgG, IgM and IgG avidity) for *T. gondii*.^39^

The *T. gondii* IgG avidity test is useful to distinguish past infection from present infection in specific IgM-positive pregnant women. In a study in Brazil, the presence of anti-*T. gondii* IgM in an immune pregnant woman was often not associated with congenital infection. Twenty per cent of infants born to immune mothers with specific IgM antibodies and low avidity IgG antibodies were vertically infected, while 8% of infants born to mothers with specific IgM antibodies and intermediate or high IgG avidity were vertically infected.^40^ Liesenfeld et al. evaluated the usefulness of testing for IgG avidity in association with *T. gondii* and found that among pregnant women with either positive or equivocal specific IgM, 55.9% had high avidity-specific IgG.

### Prevention strategy

The high rate of *T. gondii* susceptibility among pregnant women could justify health education interventions. Women of childbearing potential found to be susceptible to *T. gondii* should be educated on behaviour to prevent infection.^41^ A health promotion strategy should be aimed at women of reproductive age, making them aware of preventive behaviour.^38^

## Conclusion

The study group of urban pregnant women attending public antenatal care in Windhoek from September to October 2016 had a low prevalence of antibodies to *T. gondii*. This could possibly be attributed to geographical and meteorologic aspects. The youngest and the oldest age groups were least affected by toxoplasmosis. Health education of pregnant women on preventive measures could minimise the risk of CT. Further studies could investigate the seroprevalence of *T. gondii* in rural areas of Namibia, where higher rates of infection might be found. Future investigations could also be directed towards a one health approach to focus on *T. gondii* infection among wildlife-like game and carnivores and among abattoir workers. Of interest is the lack of information on genotypes of *T. gondii* found in Southern Africa.

## References

[1] Remington JS, McLeod R, Wilson CB, Desmonts G. Toxoplasmosis. In: Remington JS, Klein JO, Wilson CB, Nizet V, Maldonado YA, editors. Infectious diseases of the fetus and newborn infant. 7th ed. Philadelphia, PA: Saunders, 2011; p. 918–1041.

[2] Da Silva MG, Vinaud MC, De Castro AM. Prevalence of toxoplasmosis in pregnant women and vertical transmission of *Toxoplasma gondii* in patients from basic units of health from Gurupi, Tocantis, Brazil, from 2012 to 2014. PLoS One. 2015;10(11):e0141700. 10.1371/journal.pone.0141700 26558622 PMC4641701

[3] Oz HS. Maternal and congenital toxoplasmosis, currently available and novel therapies on horizon. Front Microbiol. 2014;5:Article 385. 10.3389/fmicb.2014.00385 PMC4109466 25104952

[4] Fernandes MA, Batista GI, Carlos JS, et al. *Toxoplasma gondii* antibody profile in HIV-1-infected and uninfected pregnant women and the impact on congenital toxoplasmosis diagnosis in Rio de Janeiro, Brazil. Braz J Infect Dis. 2012;16(2):170–174. 10.1016/S1413-8670(12)70300-8 22552460

[5] Montoya JG, Liesenfeld O. Toxoplasmosis. Lancet. 2004 6 12;363:1965–1976. 10.1016/S0140-6736(04)16412-X 15194258

[6] Wilson CB, Remington JS, Stagno S, Reynolds DW. Development of adverse sequelae in children born with subclinical congenital toxoplasmosis infection. Pediatrics. 1980;66(5):767–774. 7432882

[7] Lappalainen M, Koskineimi M, Hiilesmaa V, et al. Outcome of children after maternal primary *Toxoplasma* infection during pregnancy with emphasis on avidity of specific IgG. The Study Group. Pediatr Infect Dis J. 1995;14(5):354–361. 10.1097/00006454-199505000-00004 7638009

[8] Jacobs MR, Mason PR. Prevalence of *Toxoplasma* antibodies in Southern Africa. S Afr Med J. 1978;53:619–621. 675435

[9] Van der Colf BE, Noden BH, Wilkinson R, Chipare I. Low seroprevalence of antibodies to *Toxoplasma gondii* in blood donors in central Namibia. S Afr J Infect Dis. 2014;29(3):101–104. 10.1080/23120053.2014.11441579

[10] Development Core Team. R: A language and environment for statistical computing [homepage on the Internet]. Vienna: R Foundation for Statistical Computing; 2008 [cited 2017 Dec 04]. ISBN 3-900051-07-0. Available from: http://www.R-project.org

[11] Alvarado-Esquivel C, Sifuentes-Alvarez A, Narro-Duarte SG, et al. Seroepidemiology of *Toxoplasma gondii* infection in pregnant women in a public hospital in northern Mexico. BMC Infect Dis. 2006;6;Article 113. 10.1186/1471-2334-6-113 PMC1543640 16839423

[12] Hammond-Aryee K, Esser M, Van Helden PD. *Toxoplasma gondii* seroprevalence studies on humans and animals in Africa. S Afr Fam Pract. 2014;56(2):119–124. 10.1080/20786204.2014.10855349

[13] Abdel-Hameed AA. Sero-epidemiology of toxoplasmosis in Gezira, Sudan. J Trop Med Hyg. 1991;94(5):329–332. 1942211

[14] Rodier MH, Berthonneau J, Bourgoin A, et al. Seroprevalence of toxoplasma, malaria, rubella, cytomegalovirus, HIV and treponemal infections among pregnant women in Cotonou, Republic of Benin. Acta Tropica. 1995 4 8;59(4):271–277. 10.1016/0001-706X(95)00087-U 8533662

[15] Doehring E, Reiter-Owona I, Bauer O, et al. *Toxoplasma gondii* antibodies in pregnant women and their newborns in Dar es Salaam, Tanzania. Am J Trop Med Hyg. 1995;52(6):546–548. 10.4269/ajtmh.1995.52.546 7611563

[16] Singh S, Pandit AJ. Incidence and prevalence of toxoplasmosis in Indian pregnant women: A prospective study. Am J Reproduct Immunol. 2004 10;52(4):276–283. 10.1111/j.1600-0897.2004.00222.x 15494049

[17] Negash T, Tilahun G, Medhin G. Seroprevalence of *Toxoplasma gondii* in Nazareth Town, Ethiopia. Cent Afr J Med. 2007;53(9–12):47–51. 10.4314/cajm.v53i9-12.62616 20353125

[18] Uneke CJ, Duhlinska DD, Ngwu BA, Njoku MO. Seroprevalence of *Toxoplasma gondii* infection in Kwal, a rural district of Plateau-Nigeria. Afr J Med Sci. 2007;36(2):109–113. 19205571

[19] Swai ES, Schoonman L. Seroprevalence of *Toxoplasma gondii* infection among residents of Tanga district in north-east Tanzania. Tanzania J Health Res. 2009;11(4):205–209. 10.4314/thrb.v11i4.50178 20734700

[20] Dos Santos Goncalves MA, Brandao De Matos CC, Spegiorin LCJF, Vaz-Oliani DCM, De Mattos LC. Seropositivity rates for toxoplasmosis, rubella, syphilis, cytomegalovirus, hepatitis and HIV among pregnant women receiving care at a public health service, Sao Paulo State, Brazil. Braz J Infect Dis. 2010;14(6):601–605. 10.1016/S1413-8670(10)70118-5 21340301

[21] Mickoto BM, Akue JP, Bisvigou U, Tsonga SM, Nkoghe D. Serological study on toxoplasmosis among pregnant women from Franceville, Gabon. Bull La Soc Pathol Exot. 2010;3(1):41–43. 10.1007/s13149-009-0031-6 20084487

[22] Sitoe SPBL, Rafael B, Meireles LR, Andrade HF, Jr, Thompson R. Preliminary report of HIV and *Toxoplasma gondii* occurrence in pregnant women from Mozambique. Rev Inst Med Trop Sao Paulo. 2010 12;52(6):291–295. 10.1590/S0036-46652010000600002 21225211

[23] Kistiah K, Barragan A, Winiecka-Krusnell J, Karstaedt A, Frean J. Seroprevalence of *Toxoplasma gondii* in HIV-positive and HIV-negative subjects in Gauteng, South Africa. S Afr J Epidemiol Infect. 2011;26(4, Part I):225–228. 10.1080/10158782.2011.11441457

[24] Deji-Agboola AM, Busari OS, Osinupebi OA, Amoo AO. Seroprevalence of *Toxoplasma gondii* antibodies among pregnant women attending antenatal clinic of Federal Medical Center, Lagos, Nigeria. Int J Biol Med Res. 2011;2(4):1135–1139.

[25] El Deeb HK, Salah-Eldin H, Khodeer S, Abdu Allah A. Prevalence of *Toxoplasma gondii* infection in antenatal population of Menoufia governate, Egypt. Acta Tropica. 2012;124:185–191. 10.1016/j.actatropica.2012.08.005 22921952

[26] De Paschale M, Ceriani C, Cerulli T, et al. Antenatal screening for *Toxoplasma gondii*, cytomegalovirus, rubella and *Treponema pallidum* infections in northern Benin. Trop Med Int Health. 2014 6;19(6):743–746. 10.1111/tmi.12296 24612218

[27] Doudou Y, Renaud P, Coralie L, et al. Toxoplasmosis among pregnant women: High seroprevalence and risk factors in Kinshasa, Democratic Republic of Congo. Asian Pac J Trop Biomed. 2014;4(1):69–74. 10.1016/S2221-1691(14)60211-2 24144134 PMC3819499

[28] Awoke K, Nibret E, Munshea A. Sero-prevalence and associated risk factors of *Toxoplasma gondii* infection among pregnant women attending antenatal care at Felege Referral Hospital, northwest Ethiopia. Asian Pac J Trop Med. 2015;8(7):549–554. 10.1016/j.apjtm.2015.06.014 26276286

[29] Alvarado-Esquivel C, Corella-Madueno MAG, Hernandez-Tinoco J, et al. Seroepidemiology of *Toxoplasma gondii* infection in women of reproductive age: A cross-sectional study in a Northwestern Mexican city. J Clin Med Res. 2018;10(3):210–216. 10.14740/jocmr3284w 29416579 PMC5798267

[30] Neto EC, Amorim F, Lago EG. Estimation of the regional distribution of congenital toxoplasmosis in Brazil from the results of neonatal screening. Sci Med. 2010;20(1):64–70.

[31] Torgerson PR, Mastroiacovo P. The global burden of congenital toxoplasmosis: A systematic review. Bull World Health Org. 2013;91:501–508. 10.2471/BLT.12.111732 23825877 PMC3699792

[32] Magwedere K, Hemberger M, Hoffman L, Dziva F. Zoonoses: A potential obstacle to the growing wildlife industry of Namibia. Infect Ecol Epidemiol Internet. 2012;2: 1–16. 10.3402/iee.v2i0.18365 PMC3474136 23077724

[33] Smith Y, Kok OB. Faecal helminth egg and oocyst counts of a small population of African lions (*Panthera leo*) in the southwestern Kalahari, Namibia. Onderstepoort J Vet Res. 2006;73(1):71–75. 10.4102/ojvr.v73i1.171 16715880

[34] Hammond-Aryee K, Esser M, Van Helden L, Van Helden P. A high seroprevalence of *Toxoplasma gondii* antibodies in a population of feral cats in the Western Cape province of South Africa. S Afr J Infect Dis. 2015;30(4):141–144. 10.1080/23120053.2015.1107295

[35] Isaac-Renton J, Bowie WR, King A, et al. Detection of *Toxoplasma gondii* oocysts in drinking water. Appl Environ Microbiol. 1998;64:2278–2280. 10.1128/AEM.64.6.2278-2280.1998 9603850 PMC106314

[36] Benenson MW, Takafuji ET, Lemon SM, Greenup RI, Sulzer AJ. Oocyst-transmitted toxoplasmosis associated with ingestion of contaminated water. N Engl J Med. 1982;307(11):666–669. 10.1056/NEJM198209093071107 7110216

[37] Cong W, Dong X-Y, Meng Q-F, et al. *Toxoplasma gondii* infection in pregnant women: A sero prevalence and case-control study in Eastern China. BioMed Res Int. 2015;2015:Article ID 170278, 1–6. 10.1155/2015/170278 PMC4619758 26539465

[38] Mohamed K, Bahathiq A, Degnah N, et al. Detection of *Toxoplasma gondii* infection and associated risk factors among pregnant women in Makkah Al Mukarramah, Saudi Arabia. Asian Pac J Trop Dis. 2016;6(2):113–119. 10.1016/S2222-1808(15)60995-1

[39] Villard O, Cimon B, L’Ollivier C, et al. Serological diagnosis of *Toxoplasma gondii* infection. Recommendations from the French National Reference Center for Toxoplasmosis. Diagn Microbiol Inf Dis. 2016;84:22–33. 10.1016/j.diagmicrobio.2015.09.009 26458281

[40] Liesenfeld O, Montoya JG, Kinney S, Press C, Remington J. Effect of testing for IgG avidity in the diagnosis of *Toxoplasma gondii* infection in pregnant women: Experience in a US reference laboratory. J Infect Dis. 2001 4 15;183(8):1248–1253. 10.1086/319672 11262207

[41] Stillwagon E, Carrier CS, Sautter M, McLeod R. Maternal serologic screening to prevent congenital toxoplasmosis: A decision-analytic economic model. PLoS Negl Trop Dis. 2011;5(9):e1333. 10.1371/journal.pntd.0001333 21980546 PMC3181241

